# Lacune de la voûte du crâne révélatrice d'un sinus pericranii

**DOI:** 10.11604/pamj.2015.21.102.7141

**Published:** 2015-06-09

**Authors:** Mahbouba Jguirim, Wafa Chebbi

**Affiliations:** 1 Service de Rhumatologie, CHU Fattouma Bourguiba Monastir, 5000 Monastir, Tunisie; 2 Service de Médecine Interne, CHU Taher Sfar Mahdia, 5100 Mahdia, Tunisie

**Keywords:** Sinus pericranii, crâne, lacune osseuse, Sinus pericranii, skull, lacunar bone

## Image en medicine

Les lacunes de la voûte du crâne sont observées sur environ 40% des radiographies standard du crâne. Elles sont découvertes le plus souvent de façon fortuite lors d'un bilan radiologique pour sinusite ou traumatisme crânien ou de façon systématique, au cours du bilan d'extension d'hémopathies ou de cancers ostéophiles. Elles sont exceptionnellement révélatrices d'un sinus pericranii. Il s'agit d'une anomalie vasculaire rare réalisant un drainage veineux entre les systèmes extra- et intracrâniens. Le diagnostic repose sur le scanner et/ou l'IRM, qui visualisent une importante prise de contraste de la lésion. Le traitement chirurgical est assez lourd et rarement indiqué. Nous rapportons l'observation d'une patiente âgée de 53 ans, aux antécédents de carcinome basocellulaire du nez, adressée pour bilan étiologique d'une lacune du crane. Le bilan biologique (numération formule sanguine, vitesse de sédimentation, protéine C-réactive, calcémie, électrophorèse des protéines) était normal. La tomodensitométrie du massif facial objectivait une image lacunaire de 9 mm à l'emporte pièce de l'os temporal gauche. A l'imagerie par résonance magnétique, la lacune est en regard du sinus transverse gauche avec lequel elle se continue et se rehausse de la même façon que le sinus après injection de gadolinium. Le diagnostic de sinus pericranii a été retenu. Devant l'absence de plainte fonctionnelle une simple surveillance a été décidée.

**Figure 1:**
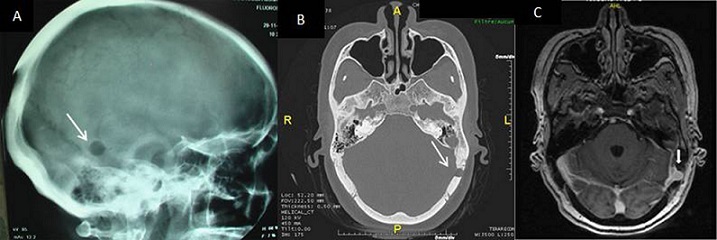
(A) radiographie du crane: lacune en regard de l'os temporal gauche; (B) TDM du massif facial: image lacunaire de 9 mm à l'emporte pièce de l'os temporal gauche; (C) IRM en coupe axiale T1 Gado: lacune en regard du sinus transverse gauche avec lequel elle se continue et se rehausse de la même façon que le sinus après injection de gadolinium

